# Comprehensive 100-bp resolution genome-wide epigenomic profiling data for the hg38 human reference genome

**DOI:** 10.1016/j.dib.2022.108827

**Published:** 2022-12-14

**Authors:** Ronnie Y. Li, Yanting Huang, Zhiyue Zhao, Zhaohui S. Qin

**Affiliations:** a Graduate program in Neuroscience, Emory University, United States; b Department of Computer Science, Emory University, United States; c Department of Biostatistics and Bioinformatics, Emory University, United States

**Keywords:** ENCODE, Genomics, Epigenomics, High-throughput sequencing, Bioinformatics, ATAC-seq, assay for transposase-accessible chromatin with sequencing, ChIP-seq, chromatin immunoprecipitation followed by sequencing, DNase-seq, DNase I hypersensitive site assay with sequencing, ENCODE, Encyclopedia of DNA Elements, EWAS, epigenome-wide association study, gnomAD, Genome Aggregation Database, GWAS, genome-wide association study, TF, transcription factor

## Abstract

This manuscript presents a comprehensive collection of diverse epigenomic profiling data for the human genome in 100-bp resolution with full genome-wide coverage. The datasets are processed from raw read count data collected from five types of sequencing-based assays collected by the Encyclopedia of DNA Elements consortium (ENCODE, http://www.encodeproject.org). Data from high-throughput sequencing assays were processed and crystallized into a total of 6,305 genome-wide profiles. To ensure the quality of the features, we filtered out assays with low read depth, inconsistent read counts, and poor data quality. The types of sequencing-based experiment assays include DNase-seq, histone and TF ChIP-seq, ATAC-seq, and Poly(A) RNA-seq. Merging of processed data was done by averaging read counts across technical replicates to obtain signals in about 30 million predefined 100-bp bins that tile the entire genome. We provide an example of fetching read counts using disease-related risk variants from the GWAS Catalog. Additionally, we have created a tabix index enabling fast user retrieval of read counts given coordinates in the human genome. The data processing pipeline is replicable for users’ own purposes and for other experimental assays. The processed data can be found on Zenodo at https://zenodo.org/record/7015783. These data can be used as features for statistical and machine learning models to predict or infer a wide range of variables of biological interest. They can also be applied to generate novel insights into gene expression, chromatin accessibility, and epigenetic modifications across the human genome. Finally, the processing pipeline can be easily applied to data from any other genome-wide profiling assays, expanding the amount of available data.

**Specifications Table**

**Table:** 

Subject	Bioinformatics
Specific subject area	High-throughput sequencing, genomic and epigenomic profiling
Type of data	Table Figure Compressed, tab-delimited read counts Tabix index of genome-wide read counts
How the data were acquired	The data were acquired first by retrieving the metadata from the ENCODE consortium (http://www.encodeproject.org). The alignment files (.bam) for the experiments of interest were downloaded and processed into .csv format as read counts. Read counts were measured in approximately 30 million pre-defined 100-bp bins across the genome.
Data format	Analyzed Filtered
Description of data collection	The merged read counts were acquired by filtering out low-quality experimental assays from the ENCODE metadata. All data with audit colors of yellow and red were excluded from processing. Python and R were used to process the metadata and alignment files. Read counts from technical replicates were merged by taking the mean read count across technical replicates in each 100-bp bin.
Data source location	**Primary data source:** ENCODE Project (http://encodeproject.org)
Data accessibility	Repository name: Zenodo Data identification number: 10.5281/zenodo.7015783 Direct URL to data: https://zenodo.org/record/7015783
Related research article	**For a published article:** Y. Huang, X. Sun, H. Jiang, S. Yu, C. Robins, M.J. Armstrong, R. Li, Z. Mei, X. Shi, E.S. Gerasimov, P.L. De Jager, D.A. Bennett, A.P. Wingo, P. Jin, T.S. Wingo, Z.S. Qin, A machine learning approach to brain epigenetic analysis reveals kinases associated with Alzheimer's disease, Nat Commun 12(1) (2021) 4472. 10.1038/s41467-021-24710-8.

**Value of the Data**

• These data provide a comprehensive measure of read counts from a well-known database of high-throughput omics data.
• Machine learning experts, computational biologists, biostatisticians, and bioinformaticians alike can benefit from these data by investigating genomic and epigenomic states at various loci.
• These data can be used as genomic and epigenomic features for machine learning and statistical models aiming to predict a range of biologically relevant variables, such as disease-associated variants.

## 1 Objective

The development of high-throughput sequencing assays like RNA-seq and ATAC-seq has enabled the generation of large numbers of “omics” datasets from a wide array of tissues and cell types, which provide insights into many traits of biological importance and make them a powerful resource for biomedical research. Nevertheless, much of these datasets are stored in formats that cannot be directly utilized by machine learning algorithms and other computational software. A significant amount of effort is required to process these data before they can be used. Here, we aimed to construct a collection of uniformly processed omics datasets consisting of the genome-wide signal, in 100-bp resolution, from a multitude of experiments extracted from the ENCODE database in a widely accessible and interpretable tabular format. We believe these carefully processed data provide the research community a useful resource to incorporate omics data in their statistical and machine learning models.

## 2 Data Description

This manuscript provides data of processed, merged read counts from high-throughput sequencing experiments obtained from the ENCODE consortium (http://www.encodeproject.org) [1]. The data can be accessed via Zenodo at https://zenodo.org/record/7015783, and the source code used to process the data can be found at https://github.com/YantingHuang/ENCODE [2].

These data were used by our group to develop computational tools for the identification of disease-specific noncoding variants (DIVAN) [3], for disease category-specific annotation of variants (CASAVA) [4], and for the prediction of novel kinases associated with Alzheimer's disease (EWASplus) [5]. Other widely used computational and machine learning tools have depended on similar types of data [6], [7], [8], [9], [10]. The total number of processed features for each sequencing-based assay is shown in Table 1. Each feature represents an individual experiment (determined by a unique ENCODE accession number) or, if applicable, a merging of the technical replicates in an individual experiment. These features constitute the columns of their respective g-zipped tab-delimited files.

**Table 1:** Number of features for each sequencing experiment already processed into its respective .tsv.gz file

Sequencing experiment type	Number of features
ATAC-seq	87
DNase-seq	822
Histone ChIP-seq	1787
TF ChIP-seq	3310
Poly-A RNA-seq	299

**Total**	**6305**

Sequencing data from five types of experiments were aggregated separately: assay for transposase-accessible chromatin (ATAC-seq; ATAC_seq_merged_counts.tsv.gz), DNase-seq (DNase_seq_merged_counts.tsv.gz), chromatin immunoprecipitation (ChIP-seq; Histone_ChIP_seq_merged_counts.tsv.gz and TF_ChIP_seq_merged_counts.tsv.gz), and RNA-seq with poly(A) capture (PolyA_RNAseq_merged_counts.tsv.gz).

Along with their respective tab-delimited files, tabix indices were also created to facilitate access to the files by allowing users to retrieve specific genomic coordinates, precluding the need to load the entire file in computer memory (ATAC_seq_merged_counts.tsv.gz.tbi, DNase_seq_merged_counts.tsv.gz.tbi, Histone_ChIP_seq_merged_counts.tsv.gz.tbi, PolyA_RNAseq_merged_counts.tsv.gz.tbi, and TF_ChIP_seq_merged_counts.tsv.gz.tbi) [11].

**Fig. 1:**
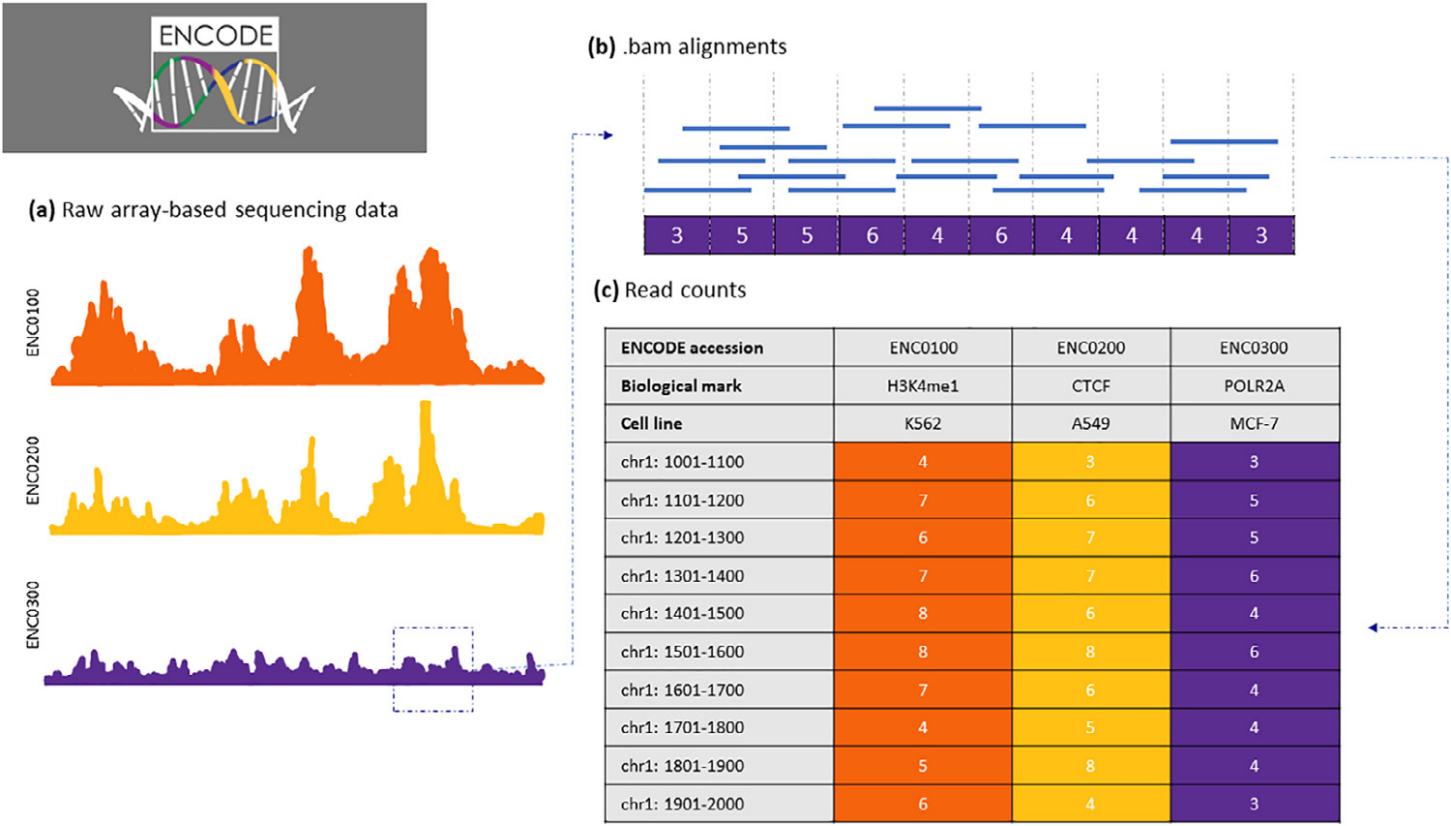
Schematic of data collection process and format of data. **(a)** Raw read counts from sequencing-based assays are imported as .bam files. **(b)** Each bam file contains a multitude of reads covering specific genomic intervals. We calculated the number of reads that overlap each pre-defined 100-bp window and saved these counts as compressed .tsv files. **(c)** Processed read counts are in tabular format, with rows representing the genomic intervals and columns constituting the experimental accession numbers. Each accession number represents the sequencing experiment of a biological target sample done in a specific cell line.

Fig. 1 illustrates a schematic of the data processing workflow. We started with the mapped reads (.bam files). Next, we calculated the number of reads that overlap our pre-defined 100-bp genomic intervals, and we tabulated the results.

## 3 Experimental Design, Materials and Methods

A general overview of the data processing pipeline, as well as the tab-delimited format of our tab-delimited data, is represented in Fig. 1.

### 3.1 Metadata acquisition and filtering

Metadata containing experiment accessions were downloaded from the official ENCODE database. We adhered to the data standards posted by ENCODE and filtered out data with audit colors of red and yellow. In doing so, this removed experiments with extremely low read depth, missing control alignments, inconsistent read counts, and other problems described in detail in https://www.encodeproject.org/data-standards/audits/. We continued to filter unwanted data based on the following self-imposed criteria: We only kept .bam files as they are already in the correct file format to be processed by our scripts. Files must have already been released and available on the ENCODE platform. Importantly, only data using human reference genome hg38 were kept, ensuring consistency among genome builds. Finally, for histone ChIP-seq data, we excluded all redacted unfiltered alignments.

### 3.2 Read count extraction and merging

Read counts were calculated from each .bam file individually by determining the number of reads that overlap within each pre-defined 100-bp interval in the genome. After downloading the .bam files, we used the R Bioconductor packages GenomicRanges and GenomicAlignments to calculate overlapping reads [12]. Due to the heavy computational burden required for this procedure, we sent all jobs to the XSEDE Comet high-performance computing cluster at the Rollins School of Public Health at Emory University, which uses Slurm to submit jobs. For each downloaded .bam file, we found the overlaps of the reads with pre-defined bins, output the read counts to a .tsv file, and deleted the .bam file.

Because many experiments in the ENCODE database utilized technical replicates, which are from the same biological sample, we decided to average the read counts for each .bam file across all its technical replicates belonging to the same experiment accession, thereby obtaining a measure of the overall signal in each bin. To obtain the list of technical replicates, we used the downloaded metadata and applied the same filtering criteria as described earlier. However, we treated biological replicates as independent observations, since they used different biological samples. Using TF ChIP-seq as an example, some columns in the resulting data matrix might contain observations from the same transcription factor in the same cell line, but from different institutions and laboratories. These are considered biological replicates and were not merged. However, if the experiments themselves were conducted on technical replicates (i.e., the same biological samples), then these read counts were merged by taking the arithmetic mean.

Since the data we provided are designed to be used as features to predict genomic characteristics such as transcription factor binding sites and mutation pathogenicity, and since comparison is done feature by feature, standardization across features is not required.

### 3.3 Example from GWAS data

To demonstrate the utility of our dataset, we provide an example in which we efficiently retrieve signals from bins near a list of GWAS variants from the GWAS Catalog [13]. First, we processed a list of risk variants and matched neutral variants based on allele frequency and genomic context from the gnomAD database. Next, we used tabix to extract the regions of interest in batch. In addition to the central 100-bp bin containing the variant, we also obtained read counts for 10 upstream and 10 downstream bins, comprising a total of 21 100-bp bins for each variant. Users can input their own genomic coordinates to retrieve signals for their own purposes.

The utility of our data lies in the fact that it provides a more informative measure of the signal at a genomic locus compared to typical datasets from ENCODE, which are binary signals indicating whether there is a sequencing peak at a locus. In our recent paper, we implemented an ensemble method called DIVAN to identify disease-specific noncoding variants [3]. Indeed, we showed that a top feature distinguishing benign SNPs from disease-associated SNPs was a closed chromatin mark, H3K9me3. Existing methods were not able to detect this informative feature because they used binary indicators showing only peak presence or absence at a particular locus. Using a more continuous measure of read counts enabled greater sensitivity for detection and resulted in more informative features being included in the final model.

### 3.4 Data and code availability

All data can be accessed via Zenodo at https://zenodo.org/record/7015783, and the source code can be found at https://github.com/YantingHuang/ENCODE.

## Ethics Statements

This work does not contain any studies with human or animal subjects.

## CRediT authorship contribution statement

**Ronnie Y. Li:** Data curation, Writing – original draft, Validation. **Yanting Huang:** Data curation, Conceptualization, Methodology, Software. **Zhiyue Zhao:** Data curation, Validation. **Zhaohui S. Qin:** Supervision, Writing – review & editing.

## Declaration of Competing Interest

The authors declare that they have no known competing financial interests or personal relationships that could have appeared to influence the work reported in this paper.

## Data Availability

Comprehensive 100-bp resolution genome-wide epigenomic profiling data for the hg38 human reference genome (Original data) (Zenodo).

## References

[1] Consortium The ENCODE Project (2012). An integrated encyclopedia of DNA elements in the human genome. Nature.

[2] R. Li, Y. Huang, Z.S. Qin, Comprehensive 100-bp resolution genome-wide epigenomic profiling data for the hg38 human reference genome, V1.0, 2022[dataset]. doi:10.5281/zenodo.7015783. PMC9792340 36582986

[3] Chen L., Jin P., Qin Z.S. (2016). DIVAN: accurate identification of non-coding disease-specific risk variants using multi-omics profiles. Genome Biol..

[4] Cao Z., Huang Y., Duan R., Jin P., Qin Z.S., Zhang S. (2022). Disease category-specific annotation of variants using an ensemble learning framework. Brief Bioinform..

[5] Huang Y., Sun X., Jiang H., Yu S., Robins C., Armstrong M.J., Li R., Mei Z., Shi X., Gerasimov E.S., De Jager P.L., Bennett D.A., Wingo A.P., Jin P., Wingo T.S., Qin Z.S. (2021). A machine learning approach to brain epigenetic analysis reveals kinases associated with Alzheimer's disease. Nat. Commun..

[6] Rentzsch P., Witten D., Cooper G.M., Shendure J., Kircher M. (2019). CADD: predicting the deleteriousness of variants throughout the human genome. Nucleic. Acids. Res..

[7] Lu Q., Hu Y., Sun J., Cheng Y., Cheung K.-H., Zhao H. (2015). A statistical framework to predict functional non-coding regions in the human genome through integrated analysis of annotation data. Sci. Rep..

[8] Ritchie G.R.S., Dunham I., Zeggini E., Flicek P. (2014). Functional annotation of noncoding sequence variants. Nat. Methods.

[9] Zhou L., Zhao F. (2018). Prioritization and functional assessment of noncoding variants associated with complex diseases. Genome Med..

[10] Ionita-Laza I., McCallum K., Xu B., Buxbaum J.D. (2016). A spectral approach integrating functional genomic annotations for coding and noncoding variants. Nat. Genet..

[11] Li H. (2011). Tabix: fast retrieval of sequence features from generic TAB-delimited files. Bioinformatics.

[12] Lawrence M., Huber W., Pagès H., Aboyoun P., Carlson M., Gentleman R., Morgan M.T., Carey V.J. (2013). Software for computing and annotating genomic ranges. PLoS Comput. Biol..

[13] Buniello A., Jacqueline A., Cerezo M., Harris L.W., Hayhurst J., Malangone C., McMahon A., Morales J., Mountjoy E., Sollis E., Suveges D., Vrousgou O., Whetzel P.L., Amode R., Guillen J.A., Riat H.S., Trevanion S.J., Hall P., Junkins H., Flicek P., Burdett T., Hindorff L.A., Cunningham F., Parkinson H. (2019). The NHGRI-EBI GWAS catalog of published genome-wide association studies, targeted arrays and summary statistics 2019. Nucleic. Acids. Res..

